# Osimertinib: A Novel Therapeutic Option for Overcoming T790M Mutations in Non–Small Cell Lung Cancer

**DOI:** 10.6004/jadpro.2017.8.2.7

**Published:** 2017-03-01

**Authors:** Shan Li, Eve M. Segal

**Affiliations:** University of Washington Medical Center/Seattle Cancer Care Alliance, Seattle, Washington

Lung cancer is the leading cause of cancer death for men and women in the United States. A total of 224,390 new cases of lung cancer and 158,080 deaths were estimated to have occurred in 2016. Furthermore, one in four deaths due to cancer is expected to be from lung cancer (Siegel, Miller, & Jemal, 2016). Despite this high morbidity and mortality, tremendous advancements have been made in the treatment of lung cancer with the use of novel targeted therapies (Johnson, Schiller, & Bunn, 2014).

Non–small cell lung cancer (NSCLC) accounts for 85% to 90% of all primary lung cancers, and a significant proportion of patients present with metastatic disease at diagnosis (Reck et al., 2014). Several genetic aberrations for the histologic subtype adenocarcinoma have been identified and are known to increase tumorigenesis and early carcinogenesis; therefore, molecular profiling of tumor samples is now considered to be the standard of care (Siegelin & Borczuk, 2014).

The most common driver mutations associated with adenocarcinoma are epidermal growth factor receptor (*EGFR*) mutations (Midha, Dearden, & McCormack, 2015). Historically, chemotherapy has been the standard in first-line therapy for metastatic NSCLC, but with limited overall effectiveness (Scagliotti et al., 2002). However, the approval of tyrosine kinase inhibitors (TKIs) such as gefitinib (Iressa), erlotinib (Tarceva), and afatinib (Gilotrif) have yielded significant increased response rates, quality of life, and fewer toxicities compared with chemotherapy (Mok et al., 2009; Rosell et al., 2012; Sequist et al., 2013; Burotto, Manasanch, Wilkerson, & Fojo, 2015). Therefore, these treatments are recommended as initial therapy in patients whose tumors harbor sensitizing *EGFR* mutations (National Comprehensive Cancer Center, 2017).

Patients who initially respond to the TKIs invariably develop biologic resistance after 8 to 16 months of therapy (Gao & Costa, 2016; Yu et al., 2013). The most common mechanism of acquired resistance is through the *EGFR* T790M mutation, which is seen in up to 50% to 60% of resistant cases (Yu et al., 2013; Peters, Zimmermann, & Adjei, 2014). Once T790M resistance emerges, the median survival is less than 2 years (Yu et al., 2013; Sequist et al., 2011).

Third-generation EGFR inhibitors such as osimertinib (Tagrisso; also known as AZD9291) have demonstrated the ability to block the growth of T790M-positive tumors and display an increased affinity for *EGFR* mutations compared with wild-type *EGFR* (Sequist et al., 2011; Tan, Gilligan, & Pacey, 2015; Liao, Lin, & Yang, 2015; Cheng, Nair, & Murray, 2016). Osimertinib was granted accelerated approval by the US Food and Drug Administration (FDA) in 2015 for the treatment of patients with advanced and metastatic *EGFR* T790M–mutant NSCLC who experienced disease progression on prior TKI therapy.

## PHARMACOLOGY AND MECHANISM OF ACTION

Osimertinib is a novel irreversible TKI with a greater affinity for mutant EGFR than wild-type EGFR (Greig, 2016). The discovery process for osimertinib was as novel as the agent itself. A systematic approach of a compound generation was employed using structure-based design for irreversible inhibitors and property-based evolution to determine kinase selectivity (Yver, 2016). When osimertinib was evaluated in preclinical trials, the agent demonstrated potent inhibition of EGFR and T790M signaling pathways in vitro and sustained tumor regression in vivo (Yver, 2016).

Osimertinib belongs to a class of small-molecule TKIs that irreversibly bind to various intracellular tyrosine kinase domains of receptors. Unlike other EGFR TKIs, osimertinib is structurally different and inhibits not only sensitizing *EGFR* mutations, but also HER2, HER3, HER4, ACK1, and BLK (AstraZeneca, 2016). Intracellular binding of osimertinib prevents tyrosine kinase activation and, therefore, inhibits EGFR downstream signaling pathways, which results in a decrease in angiogenesis and cancer-cell proliferation (Harari, 2004; Salomon, Brandt, Ciardiello, & Normanno, 1995).

## CLINICAL TRIALS

The promising evidence from preclinical research on osimertinib paved the way for further clinical trials. The phase I/II dose-escalation AURA trial evaluated the safety, efficacy, and tolerability of osimertinib in patients with advanced *EGFR*-mutated, T790M-positive NSCLC who received prior EGFR TKI therapy. The phase I portion of the trial enrolled 31 patients to receive osimertinib ranging from 20 to 240 mg/day. A total of 51% of patients experienced a partial or complete response (n = 1), 33% had stable disease, and 14% had progressive disease; the objective response rate (ORR) was 67% (95% confidence interval [CI]: 52%–70%). The maximum tolerated dose was not reached, but higher doses were associated with more side effects. Therefore, 80 mg daily was recommended for future trials (Jänne et al., 2015).

In April 2014, the FDA granted osimertinib breakthrough therapy designation following evidence from the phase I AURA trial. Subsequently, two phase II trials were developed. The AURA extension trial evaluated an additional 222 patients (n = 253) to determine ORR. The ORR was 61% (78 of 127; 95% CI: 52%–70) for those with *EGFR* T790M–mutant NSCLC, and the median duration of progression-free survival (PFS) was 9.6 months (95% CI: 8.3 to not reached; Jänne et al., 2015).

The phase II AURA2 was a multicentered, open-label, single-arm trial of 210 patients with T790M mutations who received osimertinib at 80 mg daily. After a median follow-up of 13 months, the ORR was 70%. Six patients experienced a complete response, and 67% of patients achieved a partial response. The rate of stable disease at 6 weeks of treatment was 21%, and the disease control rate for these patients was 92%. The median PFS was 9.9 months (Goss et al., 2016). These data contributed to the FDA approval of osimertinib for the treatment of patients with metastatic NSCLC who have *EGFR* T790M–mutant disease and whose disease progressed following EGFR inhibitor therapy.

A recent phase III randomized trial compared osimertinib with platinum-pemetrexed (Alimta) chemotherapy in 419 patients with *EGFR* T790M–positive metastatic NSCLC. The trial demonstrated that osimertinib improved PFS compared with chemotherapy (10.1 vs. 4.4 months; hazard ratio, 0.3; 95% CI: 0.23–0.41; p < .001). The ORR was also significantly improved with osimertinib (71%; 95% CI: 65%–76%) than with cytotoxic chemotherapy (31%; 95% CI: 24%–40%; Mok et al., 2017).

## ADVERSE EVENTS

The most common adverse events among patients receiving osimertinib were diarrhea, rash, dry skin, and nail toxicity. Grade 3 adverse effects were rather minimal (AstraZeneca, 2016; Table 1). Osimeritinib is associated with several serious side effects that require vigilant monitoring. They include cardiotoxicities, such as QTc prolongation and cardiomyopathy, and pulmonary toxicities, such as interstitial lung disease (ILD) and pneumonitis (AstraZeneca, 2016). Additionally, unlike the first- and second-generation EGFR TKIs, osimertinib was associated with neutropenia, which was reported in approximately 5% of patients in the AURA3 trial (Mok et al., 2017). It is imperative that advanced practitioners (APs) monitor patients for these unique and rare toxicities.

**Table 1:**
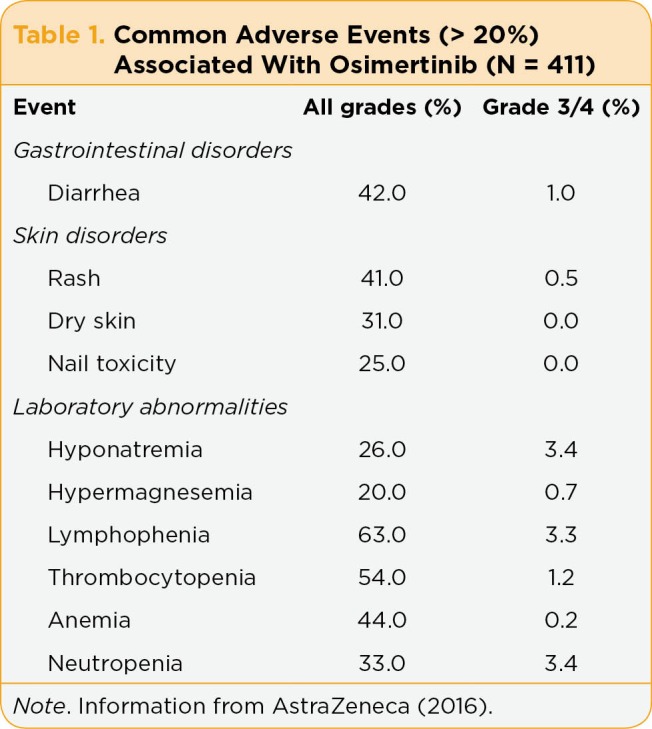
Common Adverse Events (> 20%) Associated With Osimertinib (N = 411)

## DOSING AND ADMINISTRATION

Osimertinib is commercially available in 40-mg and 80-mg tablets, and the recommended starting dose is 80 mg once daily until disease progression or unacceptable toxicity. Doses may be reduced to 40 mg for certain toxicities. Osimertinib can be administered with or without food, and absorption is not affected by agents that alter gastric acid.

In patients with difficulty swallowing tablets, osimertinib can be dispersed in 2 ounces of noncarbonated water and gently stirred. Once the tablet is in smaller pieces, patients should drink the suspension immediately. Patients should know that the tablet will not completely dissolve. Following administration, patients should add additional water into the container and drink the mixture to ensure a full dose of osimertinib was taken (AstraZeneca, 2016).

Dose adjustments are not recommended for patients with mild or moderate renal impairment. No clinically significant difference in metabolism was observed in patients with mild hepatic impairment. Osimertinib does interact with strong CYP3A inducers, and concomitant administration should be avoided. If therapy with strong CYP3A inducers cannot be avoided, the dose of osimertinib should be increased to 160 mg daily. Osimertinib can increase the exposure of medications that utilize the BCRP substrate, which will potentially increase the risk of exposure-related toxicities with these medications. Providers should monitor for adverse reactions with medications that use the BCRP substrate (AstraZeneca, 2016).

## IMPLICATIONS FOR THE ADVANCED PRACTITIONER

It is imperative for APs to provide adequate counseling for patients prior to starting osimertinib. Advanced practitioners should obtain a baseline electrocardiogram (ECG) and monitor electrolytes in patients with a history of or predisposition to QTc prolongation or for those who are on concomitant QTc-prolonging medications. Additionally, APs should note that patients with a baseline QTc of 470 msec or longer were excluded from studies with osimertinib. Per manufacturer recommendations, patients also should receive a baseline scan to assess left-ventricular ejection fraction, and subsequent scans should be performed every 3 months (AstraZeneca, 2016). Patients should also be educated about the signs and symptoms of ILD/pneumonitis and promptly evaluated for new or worsening respiratory symptoms.

Advanced practitionerts should be vigilant that patients are not currently taking strong CYP3A inhibitors or inducers while taking osimertinib. If concomitant therapy with CYP3A inhibitors is necessary, patients should be closely monitored for toxicities (AstraZeneca, 2016).

Osimertinib is associated with embryofetal toxicity. Men and women of childbearing potential should use effective contraception during treatment with osimertinib. Women should continue to use contraception for at least 6 weeks after the final dose of osimertinib, and men should use effective contraception for at least 4 months afterward (AstraZeneca, 2016).

Approximately 4.4% of patients who received osimertinib on clinical trials required dose reductions. The most frequent adverse effects that resulted in a dose reduction were QTc prolongation and neutropenia (AstraZeneca, 2016). Therefore, periodic laboratory monitoring as well as ECG in patients at risk for QTc prolongation is recommended. Table 2 illustrates the recommended dose adjustments for cardiotoxicity, pulmonary toxicity, and other grade 3 or higher adverse reactions.

**Table 2:**
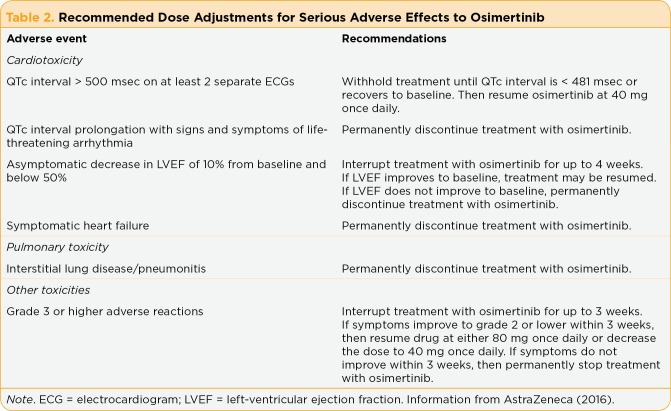
Recommended Dose Adjustments for Serious Adverse Effects to Osimertinib

## ONGOING TRIALS

Osimertinib is currently being evaluated in a variety of settings, such as first-line monotherapy for metastatic NSCLC and in combination with immunotherapy and other targeted agents (Greig, 2016). Osimertinib is believed to have a benefit as initial therapy for patients with metastatic *EGFR*-mutated NSCLC, because it can delay T790M resistance.

A study of 60 patients was conducted. A total of 30 patients received osimertinib at 80 mg, and 30 patients received 160 mg daily. The ORR was 77%, with a median PFS of 19.3 months for the 160 mg dose; the median PFS for the 80-mg dose had not been reached (Ramalingam et al., 2016). Although these findings are preliminary, they remain promising for further evaluation.

## CONCLUSION

Historically, patients with metastatic NSCLC have limited treatment options and experience significant morbidity and mortality. The use of TKIs provides an improved quality of life and better clinical outcomes compared with traditional chemotherapy (Scagliotti et al., 2002). However, acquired resistance to the EGFR TKIs eventually leads to disease progression (Yu et al., 2013). The approval of osimertinib provides a new viable therapeutic option for those with *EGFR*-mutated disease that harbors the T790M mutation.

## References

[1] AstraZeneca. (2016). Tagrisso (osimertinib) tablets prescribing information. http://www.azpicentral.com/tagrisso/tagrisso.pdf.

[2] Burotto Mauricio, Manasanch Elisabet E, Wilkerson Julia, Fojo Tito (2015). Gefitinib and erlotinib in metastatic non-small cell lung cancer: a meta-analysis of toxicity and efficacy of randomized clinical trials.. *The oncologist*.

[3] Cheng Hengmiao, Nair Sajiv K, Murray Brion W (2016). Recent progress on third generation covalent EGFR inhibitors.. *Bioorganic & medicinal chemistry letters*.

[4] Gao Xin, Le Xiuning, Costa Daniel B (2016). The safety and efficacy of osimertinib for the treatment of EGFR T790M mutation positive non-small-cell lung cancer.. *Expert review of anticancer therapy*.

[5] Goss Glenwood, Tsai Chun-Ming, Shepherd Frances A, Bazhenova Lyudmila, Lee Jong Seok, Chang Gee-Chen, Crino Lucio, Satouchi Miyako, Chu Quincy, Hida Toyoaki, Han Ji-Youn, Juan Oscar, Dunphy Frank, Nishio Makoto, Kang Jin-Hyoung, Majem Margarita, Mann Helen, Cantarini Mireille, Ghiorghiu Serban, Mitsudomi Tetsuya (2016). Osimertinib for pretreated EGFR Thr790Met-positive advanced non-small-cell lung cancer (AURA2): a multicentre, open-label, single-arm, phase 2 study.. *The Lancet. Oncology*.

[6] Greig Sarah L (2016). Osimertinib: First Global Approval.. *Drugs*.

[7] Harari P M (2004). Epidermal growth factor receptor inhibition strategies in oncology.. *Endocrine-related cancer*.

[8] Jänne Pasi A, Yang James Chih-Hsin, Kim Dong-Wan, Planchard David, Ohe Yuichiro, Ramalingam Suresh S, Ahn Myung-Ju, Kim Sang-We, Su Wu-Chou, Horn Leora, Haggstrom Daniel, Felip Enriqueta, Kim Joo-Hang, Frewer Paul, Cantarini Mireille, Brown Kathryn H, Dickinson Paul A, Ghiorghiu Serban, Ranson Malcolm (2015). AZD9291 in EGFR inhibitor-resistant non-small-cell lung cancer.. *The New England journal of medicine*.

[9] Johnson David H, Schiller Joan H, Bunn Paul A (2014). Recent clinical advances in lung cancer management.. *Journal of clinical oncology : official journal of the American Society of Clinical Oncology*.

[10] Liao Bin-Chi, Lin Chia-Chi, Yang James Chih-Hsin (2015). Second and third-generation epidermal growth factor receptor tyrosine kinase inhibitors in advanced nonsmall cell lung cancer.. *Current opinion in oncology*.

[11] Midha Anita, Dearden Simon, McCormack Rose (2015). EGFR mutation incidence in non-small-cell lung cancer of adenocarcinoma histology: a systematic review and global map by ethnicity (mutMapII).. *American journal of cancer research*.

[12] Mok Tony S, Wu Yi-Long, Ahn Myung-Ju, Garassino Marina C, Kim Hye R, Ramalingam Suresh S, Shepherd Frances A, He Yong, Akamatsu Hiroaki, Theelen Willemijn S M E, Lee Chee K, Sebastian Martin, Templeton Alison, Mann Helen, Marotti Marcelo, Ghiorghiu Serban, Papadimitrakopoulou Vassiliki A (2017). Osimertinib or Platinum-Pemetrexed in EGFR T790M-Positive Lung Cancer.. *The New England journal of medicine*.

[13] Mok Tony S, Wu Yi-Long, Thongprasert Sumitra, Yang Chih-Hsin, Chu Da-Tong, Saijo Nagahiro, Sunpaweravong Patrapim, Han Baohui, Margono Benjamin, Ichinose Yukito, Nishiwaki Yutaka, Ohe Yuichiro, Yang Jin-Ji, Chewaskulyong Busyamas, Jiang Haiyi, Duffield Emma L, Watkins Claire L, Armour Alison A, Fukuoka Masahiro (2009). Gefitinib or carboplatin-paclitaxel in pulmonary adenocarcinoma.. *The New England journal of medicine*.

[14] National Comprehensive Cancer Network® (NCCN®). (2017). NCCN Clinical Practice Guidelines in Oncology: Non–small cell lung cancer. v4.2017.. https://www.nccn.org/professionals/physician_gls/pdf/nscl.pdf.

[15] Peters Solange, Zimmermann Stefan, Adjei Alex A (2014). Oral epidermal growth factor receptor tyrosine kinase inhibitors for the treatment of non-small cell lung cancer: comparative pharmacokinetics and drug-drug interactions.. *Cancer treatment reviews*.

[16] Ramalingam S, Yang J C, Lee C K, Kurata T, Kim D W, John T, Jänne P A (2016). LBA1_PR: Osmertinib as first-line treatment for EGFR mutation-positive advanced NSCLC: Updated efficacy and safety results from two phase I expansion cohorts. *Journal of Thoracic Oncology*.

[17] Reck M, Popat S, Reinmuth N, De Ruysscher D, Kerr K M, Peters S (2014). Metastatic non-small-cell lung cancer (NSCLC): ESMO clinical practice guidelines for diagnosis, treatment, and follow-up. *Annals of Oncology*.

[18] Rosell Rafael, Carcereny Enric, Gervais Radj, Vergnenegre Alain, Massuti Bartomeu, Felip Enriqueta, Palmero Ramon, Garcia-Gomez Ramon, Pallares Cinta, Sanchez Jose Miguel, Porta Rut, Cobo Manuel, Garrido Pilar, Longo Flavia, Moran Teresa, Insa Amelia, De Marinis Filippo, Corre Romain, Bover Isabel, Illiano Alfonso, Dansin Eric, de Castro Javier, Milella Michele, Reguart Noemi, Altavilla Giuseppe, Jimenez Ulpiano, Provencio Mariano, Moreno Miguel Angel, Terrasa Josefa, Muñoz-Langa Jose, Valdivia Javier, Isla Dolores, Domine Manuel, Molinier Olivier, Mazieres Julien, Baize Nathalie, Garcia-Campelo Rosario, Robinet Gilles, Rodriguez-Abreu Delvys, Lopez-Vivanco Guillermo, Gebbia Vittorio, Ferrera-Delgado Lioba, Bombaron Pierre, Bernabe Reyes, Bearz Alessandra, Artal Angel, Cortesi Enrico, Rolfo Christian, Sanchez-Ronco Maria, Drozdowskyj Ana, Queralt Cristina, de Aguirre Itziar, Ramirez Jose Luis, Sanchez Jose Javier, Molina Miguel Angel, Taron Miquel, Paz-Ares Luis (2012). Erlotinib versus standard chemotherapy as first-line treatment for European patients with advanced EGFR mutation-positive non-small-cell lung cancer (EURTAC): a multicentre, open-label, randomised phase 3 trial.. *The Lancet. Oncology*.

[19] Salomon D S, Brandt R, Ciardiello F, Normanno N (1995). Epidermal growth factor-related peptides and their receptors in human malignancies.. *Critical reviews in oncology/hematology*.

[20] Scagliotti G V, De Marinis F, Rinaldi M, Crinò L, Gridelli C, Ricci S, Matano E, Boni C, Marangolo M, Failla G, Altavilla G, Adamo V, Ceribelli A, Clerici M, Di Costanzo F, Frontini L, Tonato M (2002). Phase III randomized trial comparing three platinum-based doublets in advanced non-small-cell lung cancer.. *Journal of clinical oncology : official journal of the American Society of Clinical Oncology*.

[21] Sequist L V, Waltman B A, Dias-Santagata D, Digumarthy S, Turke A B, Fidias P, Engelman J A (2011). Genotypic and histological evolution of lung cancers acquiring resistance to EGFR inhibitors. *Science Translational Medicine*.

[22] Sequist Lecia V, Yang James Chih-Hsin, Yamamoto Nobuyuki, O'Byrne Kenneth, Hirsh Vera, Mok Tony, Geater Sarayut Lucien, Orlov Sergey, Tsai Chun-Ming, Boyer Michael, Su Wu-Chou, Bennouna Jaafar, Kato Terufumi, Gorbunova Vera, Lee Ki Hyeong, Shah Riyaz, Massey Dan, Zazulina Victoria, Shahidi Mehdi, Schuler Martin (2013). Phase III study of afatinib or cisplatin plus pemetrexed in patients with metastatic lung adenocarcinoma with EGFR mutations.. *Journal of clinical oncology : official journal of the American Society of Clinical Oncology*.

[23] Siegelin Markus D, Borczuk Alain C (2014). Epidermal growth factor receptor mutations in lung adenocarcinoma.. *Laboratory investigation; a journal of technical methods and pathology*.

[24] Siegel R L, Miller K D, Jemal A (2016). Cancer statistics, 2016. *CA: A Cancer Journal for Clinicians*.

[25] Tan C S, Gilligan D, Pacey S (2015). Treatment approaches for EGFR-inhibitor-resistant patients with non-small-cell lung cancer. *Lancet Oncology*.

[26] Yu Helena A, Arcila Maria E, Rekhtman Natasha, Sima Camelia S, Zakowski Maureen F, Pao William, Kris Mark G, Miller Vincent A, Ladanyi Marc, Riely Gregory J (2013). Analysis of tumor specimens at the time of acquired resistance to EGFR-TKI therapy in 155 patients with EGFR-mutant lung cancers.. *Clinical cancer research : an official journal of the American Association for Cancer Research*.

[27] Yver A (2016). Osimertinib (AZD9291)-a science-driven, collaborative approach to rapid drug design and development.. *Annals of oncology : official journal of the European Society for Medical Oncology*.

